# Healthcare Students on Placements: a Cyclical Quality Method for Satisfaction Assessments

**DOI:** 10.1007/s40670-020-01048-2

**Published:** 2020-09-01

**Authors:** Camille Boutillier, Luc Jeanrenaud, Jean-Luc Gilles, Laurence Bouche, Jocelyne Quillet Cotting

**Affiliations:** 1 Haute École Pédagogique du Canton de Vaud (University of Teacher Education of State of Vaud), Avenue de Cour 33, CH1014, Lausanne, Switzerland; 2 Centre Hospitalier Universitaire Vaudois (Lausanne University Hospital), Lausanne, Switzerland

**Keywords:** Assessment of satisfaction, Test development, Medical education, Practical training, Internship, Analytic scoring, Feedback, Practicality, Standardized test, Faculty development

## Abstract

The Lausanne University Hospital (CHUV, Switzerland) is a university hospital with more than 11,000 employees who perform clinical, research and teaching roles. It was ranked in March 2019 among the ten best hospitals in the world according to the magazine Newsweek. The education scheme of the CHUV includes the practical training of more than 1700 young people, in particular from the University of Applied Sciences and Arts of Western Switzerland (HES-SO). This research and development, carried out in collaboration with the University of Teacher Education of State of Vaud (UTE Vaud), has enabled the creation and implementation of a cyclical method for evaluating the satisfaction of HES-SO healthcare students at CHUV. The method created and tested—the Cycle of Construction and Quality Control for Satisfaction Evaluations (CCQCSE)—comprises nine stages: Issues, Analysis, Design, Items, Information, Collection, Processing, Feedback and Adjustment. It was designed with the help of a multidisciplinary team of healthcare professionals (CHUV) and experts in education sciences (UTE Vaud) as part of a master’s thesis (Kaeser 2018) directed by our research team. A 4-month pilot phase allowed more than 250 placement students’ satisfaction levels to be collected with respect to ten aspects of the practical training scheme, and provided diagnostic feedback. Identifying strengths and areas for improvement has significantly helped with the development of a quality policy for student training at the institution. CCQCSE is now considered as a robust method in line with the quality measures already in place at CHUV. In particular, it encouraged those responsible for the practical training to reflect on their mentoring practices. Beyond the context of CHUV and the mentoring of healthcare students on placements, the CCQCSE model could be transferred to other sectors in which training placements are offered, opening up new avenues of collaborative research.

**Electronic supplementary material:**

The online version of this article (10.1007/s40670-020-01048-2) contains supplementary material, which is available to authorized users.

## Introduction

The Lausanne University Hospital (CHUV) is a university hospital with more than 11,000 employees who perform clinical, research and teaching roles. It was ranked in March 2019 among the ten best hospitals in the world according to the magazine Newsweek^1^. Teaching activities include the training of more than 1700 young people, in particular from the University of Applied Sciences and Arts of Western Switzerland (HES-SO) and vocational colleges in western Switzerland. Over the past few years, the education scheme set up by CHUV has been undergoing a constant process of reinforcement, expansion and professionalisation. Currently, more than 200 trainer practitioners are involved in the practical training. These placement mentors from healthcare teams have taken a teacher training course (25 days). The HES-SO students under their responsibility receive direct mentoring as part of a skills-based approach. Their practical training includes cultivating a reflective attitude. Placements are designed to be integrative (combining theory and practice).

Two years ago, efforts began on restructuring the practical training scheme at CHUV with a view to improving the quality of placements and mentoring practices. The results of the satisfaction surveys, put together using the method developed in our research, yield valuable insights for the staff of the healthcare units involved in the student training, the coordinators of the practical training who prioritise areas for development, and the managers who decide on the specific details of the restructuring operation [1].

### Literature Review and Theoretical Framework

The Kirkpatrick model [2–4] is one of the most commonly used models in the area of adult training [5]. It is based on four levels of training evaluation: reaction in terms of participant satisfaction (level 1); learning in terms of knowledge, skills or attitudes acquired and developed (level 2); transfer of the acquired knowledge, skills or attitudes from the previous level to working roles (level 3); and impact of the training on the company or organisation (level 4). Gilibert and Gillet [6] give various reasons why this four-level training evaluation design is particularly popular:

This model has the advantage of summarising the complex process of training evaluation and offers a rational approach that is sufficiently broad (but concise) to be in line with the needs of training professionals [7]. It is the most widely used model among training professionals, but also among researchers working on the evaluation of training initiatives [8].

Despite having certain weaknesses according to the literature [9–12], the Kirkpatrick model remains a benchmark, and level 1, “Reaction/satisfaction”, continues to be the most measured level in the area of training, in particular for reasons of ease, as stated by Beech and Leather:

Without doubt, the model of training evaluation by Kirkpatrick [2, 3] is still the most influential and commonly used [10, 13]. It is credited with revolutionizing the thinking on training course evaluation and remains the only model that many organizations and training departments are aware of (...). The Reaction level considers the immediate subjective opinions of participants about a course (...). Reactions are comparatively easy to measure so tend to be the first (and too often only) resort. ([14], p. 35).

Despite the popularity of the model and the widespread use of level 1 evaluations, it is worth examining the quality and validity of the information collected during these evaluations, especially given that in the current system of organising training courses in accordance with quality standards, the purpose of conducting evaluations is to improve quality of service. In this context, it is therefore entirely in the interest of stakeholders to base their adjustment and improvement decisions on reliable information. With this in mind, we have used the PTA (Parametrisation of Teaching Actions) model [18], which covers ten typical aspects of teaching-learning sequences and frameworks identified in literature specific to the field:

[…] scientific literature is abundant and there are meta-analyses examining the results of a large amount of research which point to a series of approved ‘best practices’. More recently, teams of researchers condensed the results of this contemporary research on teacher knowledge [15, 16] in order to help teaching professionals in their decision-making. (...) Over the last decade, outreach efforts have been undertaken with the aim of disseminating the results of these meta-analyses among teachers [17]. In this regard, our goal is to facilitate the adoption of a large number of effective practices identified by research, by linking them to ten interacting areas of the Parametrisation of Teaching Actions model. ([18], p. 2).

In the context of our research, the PTA model has allowed an analysis to be carried out of all the components of the practical training received by students on placements at CHUV, and has led to the design of an organisational model: the Practical Training Scheme (PTS).

In terms of the reliability of the evaluation framework used for this research, we were inspired by the Cycle of Construction and Quality Control for Standardised Testing (CCQCST) model ([19, 20], pp. 74-92). Originally, the CCQCST was developed for the purpose of preparing evaluation tools for Kirkpatrick’s level 2, “Learning”, with a focus on quality checks that make it possible to adjust and improve the products (table of specifications, evaluation plan, tools, items, documents, etc.) prepared in the process of constructing the satisfaction evaluation. A series of criteria, such as validity, reliability, feasibility, fairness and so on, enable the quality of all the elements produced. These criteria are based on the recommendations in the Standards for Educational and Psychological Testing (AREA APA NCME [21]).

## Research Questions and Hypotheses

The following question has formed the basis of our entire research project:

*Can a quality-focused satisfaction evaluation framework that is prepared using the CCQCST model adapted to level 1 of the Kirkpatrick model, help improve the CHUV placement scheme for pre-graduate HES-SO students?*

Based on this research question, we have formulated the following hypotheses:

1. A Cycle of Construction and Quality Control for Satisfaction Evaluations (CCQCSE) can be derived from the CCQCST model by making adjustments, determined by level 1, that will differentiate this cycle from the cycle initially developed for Kirkpatrick’s level 2.
2. The satisfaction evaluation framework derived from piloting the CCQCSE at CHUV will produce valid, reliable and useful information for those involved in the practical training of placement students.
3. The satisfaction evaluation framework derived from the new CCQCSE model will enable the training of placement students in the different departments to be harmonised and regulated, thus contributing to a policy for continuously improving the quality of practical training.
4. CCQCSE model quality checks will optimise the products at the various stages of construction of the satisfaction evaluation framework, and thus generate valid, reliable and useful information that will enable those involved in the practical training to identify areas for improvement in the CHUV placement scheme and to regulate its quality.

These reflections on our questions and hypotheses have led us to focus on two main components in our research. The first concerns how to design the CCQCSE in such a way as to produce satisfaction evaluation frameworks focused on quality (H1 and H2). This first component falls under research and development. The second component relates to piloting the CCQCSE at CHUV in order to produce a specific satisfaction evaluation framework which responds to the needs of the parties involved at CHUV, and which provides them with valid, reliable and useful information enabling them to make decisions with the aim of continuously improving the practical training scheme for pre-graduate placement students (H3 and H4). This second component falls under collaborative research.

## Methodology

### Research and Development Component

Our methodological approach for the component concerning the design of the CCQCSE model falls under research and development [22–24]. We followed the conventional stages of implementation proposed by Borg and Gall [25] and reiterated by Loiselle [22] and Anadón and L'Hostie [26] to develop the CCQCSE model.

### Collaborative Research Component

The second component of this research took the form of collaborative research based on the design proposed by Desgagné, that is, “one or more researchers will join up with practitioners to examine an aspect of practice and research it jointly from the perspective of all the participants’ concerns” ([27], p. 52). Thus, our research team was formed of five sub-groups (practical training coordinators, healthcare managers, trainer practitioners and two groups of students), who were consulted in the capacity of quality controllers while each stage of the CCQCSE model was being formalised.

The population sample selected for the pilot phase of the satisfaction evaluation constructed using the CCQCSE model consists of pre-graduate HES-SO bachelor students on placements in their first, second or third year of internship in one of the following fields of study: Social work, Occupational therapy, Nutrition and dietetics, Physiotherapy, Midwifery, Nursing and Technique in Medical Radiology. This sample was assembled for stages (4) Information about the evaluation, (5) Collection of data, and (7) Feedback. During the pilot phase, the survey produced using the CCQCSE model (see Appendix 1) was issued to 324 placement students between November 2017 and January 2018. In total, 262 students responded to this survey, translating into a 78% response rate.

## Results

### Research and Development Component

**Fig. 1:**
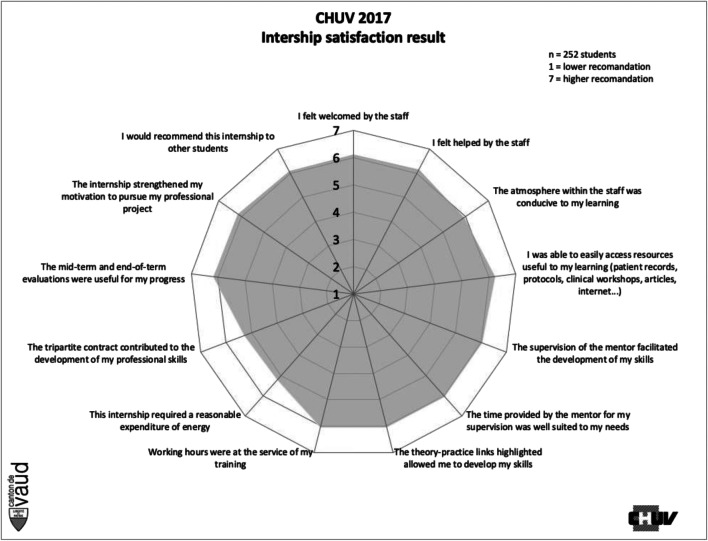
Results for the test period for the institution as a whole

The main change introduced by our research to the CCQCST model is the addition of a stage 0 entitled “Issues”. The purpose of this stage is to ensure that the evaluation framework is implemented in accordance with institutional expectations and values from an ethical point of view (Fig. 1).

This issue of guarantees offered in terms of compliance with institutional values arises not only when the work commences, but also in a cross-cutting manner throughout the implementation of the CCQCSE model. These ethical issues relate, for example, to the purposes of the evaluation, the use and anonymisation of the results, and sensitive points that need to be clarified and communicated after acceptance of the framework by the stakeholders.

A second significant change made to the CCQCST model concerns stage 1, “Analysis”, where we used the Parametrisation of Teaching Actions (PTA) model [18] as a basis for analysing all the components of the training activities designed for CHUV placement students. In addition, it has enabled targeted feedback to be provided to trainers with regard to mentoring practices.

Because of its structuring characteristics, the PTA model has enabled the “Practical Training Scheme” (PTS) model to be created, which aims to guide managers and teams at CHUV in how to improve the way student training activities are organised. The PTS adds two new aspects to those covered in the survey: “communication within the healthcare team” and “coaching for trainers”.

Taking into account the changes made to the CCQCST, we propose the following model for the CCQCSE (see Table 1).

**Table 1:**
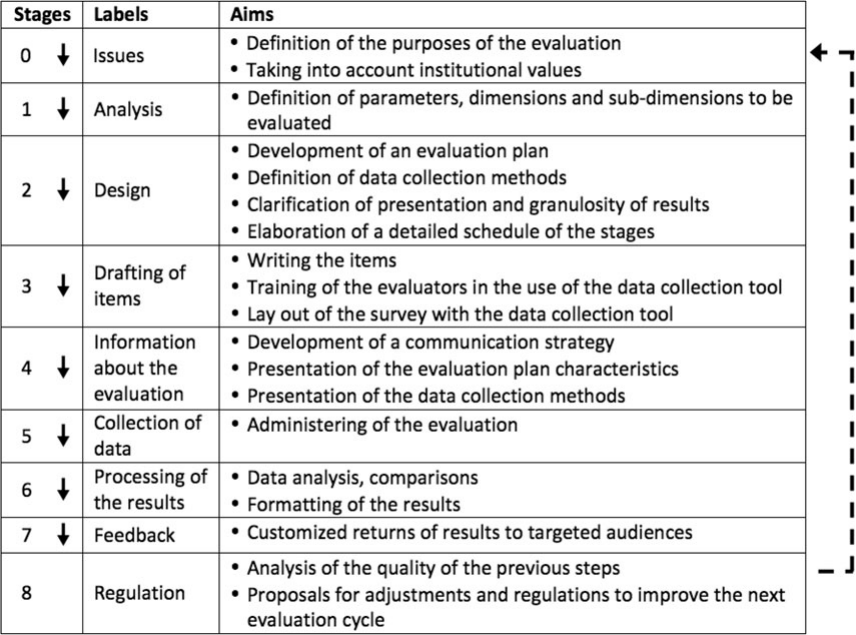
The 9-stages model of the Cycle of Construction and Quality Control for Satisfaction Evaluations (CCQCSE)

### Collaborative Research Component

In the context of the collaborative research component, quality checks were established in the first seven stages of the CCQCSE model. At each stage, the final operations and products were submitted to the members of the working group for approval and to at least one of the control groups (see Table 2). The involvement of the control groups was also intended to engage all those concerned as much as possible. This allowed for greater ownership of the model by the stakeholders.

**Table 2:** Profiles, roles controlled stages of the quality control groups

Control groups	Profiles and roles of members	Controlled stages
Body of practical training coordinators from the departments	Senior managers and nurses specialised in adult education	1 Analysis 2 Design 3 Drafting of items 4 Information about the evaluation 5 Collection of data 6 Processing of the results 7 Feedback
Healthcare managers	Head of department nurses	1 Analysis 2 Design 3 Drafting of items 4 Information about the evaluation 5 Collection of data
Trainer practitioners from the departments	Trainers supporting the students in the departments	1 Analysis 2 Design 3 Drafting of items 5 Collection of data
Students from the Haute École de la Santé La Source	Representatives of nursing students in their last year of study	1 Analysis 2 Design
Students from the Haute École de Santé Vaud (HESAV)	Representatives of nursing students in their last year of study	1 Analysis 2 Design

The quality checks also helped fuel overall reflection on the evaluation approach. This reflection concerned not only the purposes, but also the processes, and reinforced the idea of adding the prior “Issues” stage to the CQCSE cycle.

Finally, this reflection helped drive institutional efforts on the PTS and identify areas for improvement.

The data collected in stage (5), “Collection”, was processed in the following stage, (6) “Processing”, to transform it into “radar” charts, which were then transferred to stage (7), “Feedback”. The most striking aspect of these initial results was the satisfaction rate of over 80% for almost all the items. Only two items showed a satisfaction rate slightly below 80%: the performance of tripartite agreements (78%) and the energy expenditure required of placement students (77%).

**Fig. 2:**
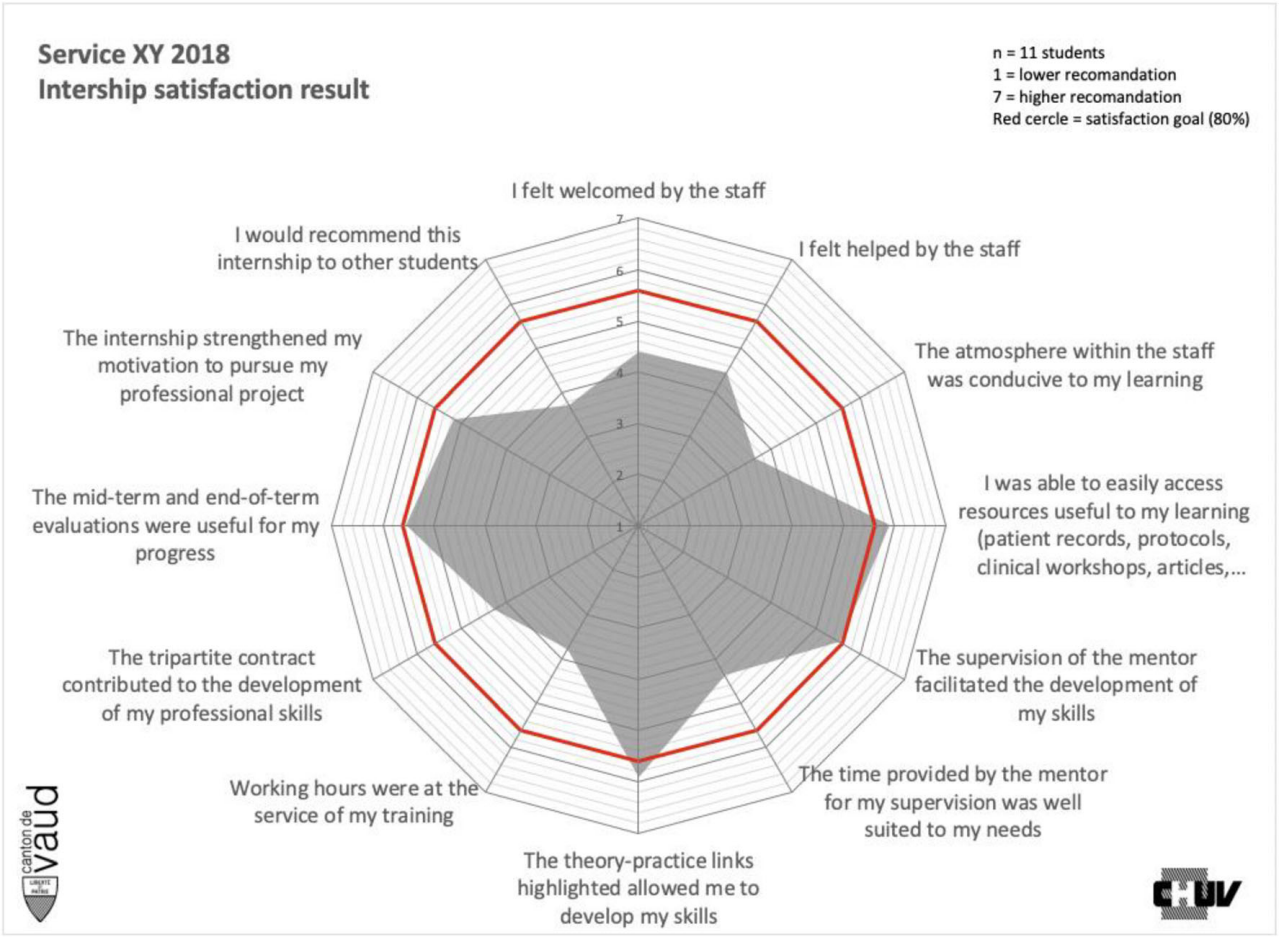
Focus on the results of the department “XY” before regulation

Each department received personalised results that included the baseline institutional value (shown in red in Fig. 2), allowing them to identify their strengths and areas for improvement. This type of feedback also makes it possible to identify departments and healthcare units that offer support practices, which are popular among placement students. These practices can then be discussed and approved for other departments and healthcare units, particularly those requiring targeted improvement. Eventually, after several satisfaction evaluation cycles, those in charge of placements will be able to measure the impact of the changes made.

### Impact on Mentoring Practices: Report on a Specific Outcome

As of January 2018, this questionnaire has been made permanent for all students in the CHUV’s health care programs.

After only 6 months, the first results identified that a particular service has a very low result for each item and worrying comments. The latter consisted of comments highlighting possible abuses by the team towards the trainees.

“There are some people who are obnoxious to students! We should review with the team the level of requirement requested according to our level of training", "There is really a big lack of respect from these people towards us, students”, “There is a big lack of respect from these people towards us, students”.

The Department of Care of the CHUV and the Department concerned were able to make the link with a training climate that is reputed to be very demanding for this service, worrying feedback from the universities about their students’ state of stress after their internship, and the writings of eight of them collected through the questionnaire. These elements led to a first session which brought together the service’s care managers and the departmental and institutional directors.

An initial observation made it possible to identify the difficulties raised in this internship had been known for a long time but that no written record had been collected. No names had ever been mentioned. The partner universities encountered exactly the same problem. Many students had come to complain after their traineeship, but none wished to leave a formal trace. This is how we discovered a real climate of omerta at the internship site.

The second step was to present the results to the service’s trainers. This was a difficult session where the actors expressed their difficulties in receiving feedback from the students.

Subsequently, further meetings were necessary to bring out a number of common concerns and problems:

1. Lack of collaboration between the Practitioner Trainers
2. Climate of Suffering of the Health Care Team
3. Senior service manager experienced by the team as little invested in training issues
4. Specific work culture tolerates a certain level of abuse in training

Finally, an action plan was put in place based on these problems with the help of all the actors involved in the service. It includes many times of activities regulation for the professional trainers but also more systemic actions on the staffing of the care team.

After 1 year, the results were evident from the students’ point of view. For the health care team, they were a source of great pride. The whole team and the management were also congratulated for the project and its excellent results.

In retrospect, it seemed to us that the visibility of the results through the feedback really raised the awareness of those involved. We hypothesize that the graphic formatting will have facilitated change among professionals

**Fig. 3:**
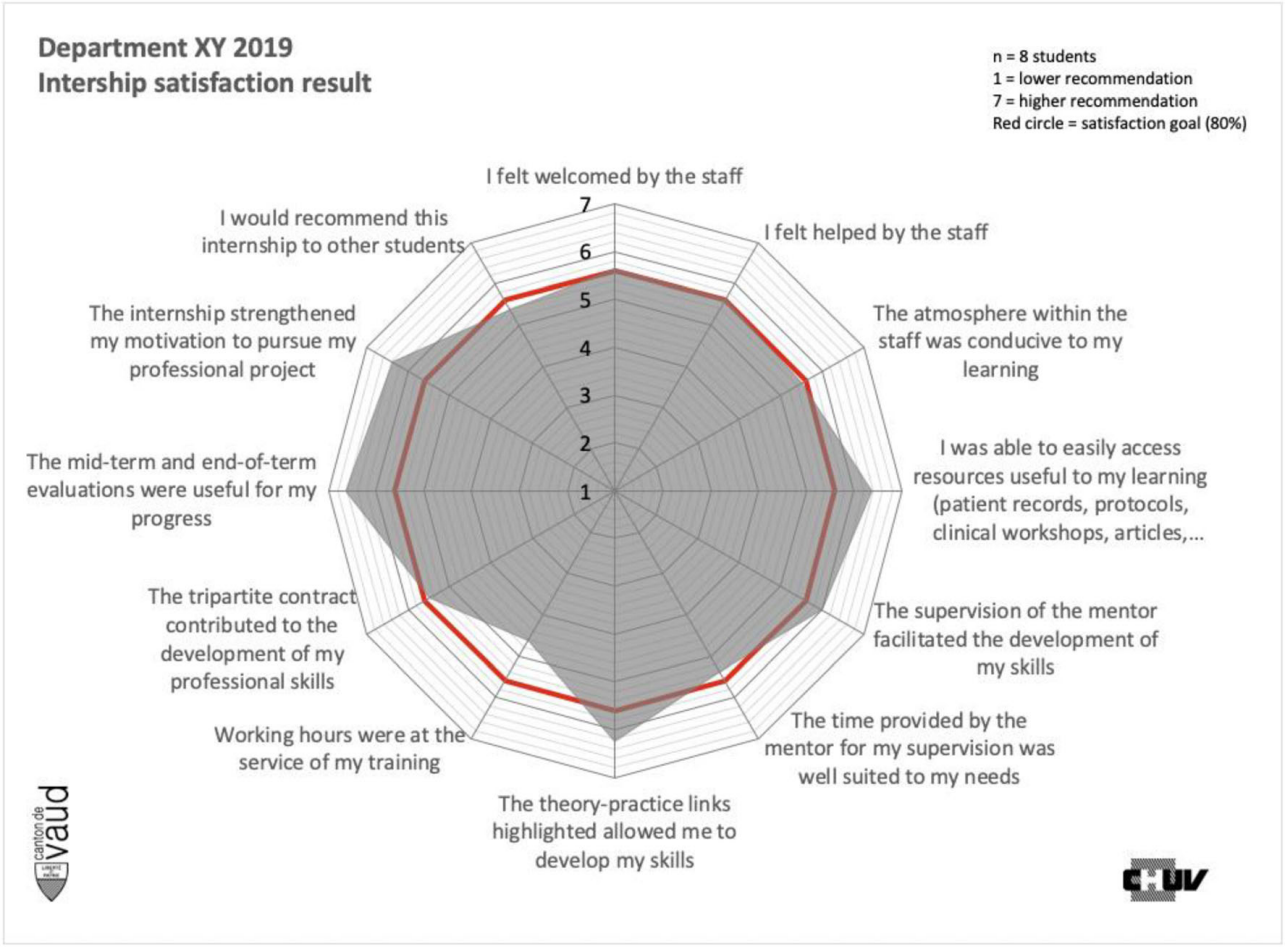
Focus on the results of the department “XY” after regulation

One of the questions is not on the graph: question on energy expenditure. It was, in fact, removed for the institution in 2019, due to a difficulty in interpreting the results. The wording of the question is currently being reviewed with a view to reintegrating this dimension (Fig. 3).

## Discussion

The approach that was implemented to construct the tool for measuring the satisfaction of placement students, using the CCQCSE model, allowed us to verify our four hypotheses.

With regard to hypothesis H1, two major adjustments were made. The first was to incorporate reflections on ethical issues into stage (0), “Issues”, particularly in terms of whether or not to use placement students’ grades when processing the results. In the end, the decision was made not to include this information.

Another notable difference that emerged concerns stage (1), “Analysis”. Specifically, the CCQCST model involves the preparation of a Table of Specifications (ToS) that clarifies the objectives to be evaluated by linking the contents of the training to the corresponding cognitive skills that are expected. In the case of the CCQCSE model, it is not a conventional ToS that is constructed, but a table setting out the aspects and sub-aspects of the satisfaction evaluation based on the Parametrisation of Teaching Actions (PTA) model.

As the stages of the cycle progressed, it became increasingly evident that the people on the ground needed preparation for receiving and incorporating the information contained in the feedback, using the PTS model. Feedback alone is not enough to change practices. For real improvement, the evaluation framework must include an organisational model (PTS) that incorporates this feedback in order to improve the efficiency of student support practices.

At the time of the pilot phase, after a 4-month period, the response rate was 78%. Almost all the respondents left detailed comments.

The extensive feedback generated by the CCQCSE proved extremely useful for improving the mentoring services of the healthcare teams at CHUV.

Firstly, implementing the CCQCSE model allowed areas of teaching activity requiring improvement to be clearly defined. Aside from the opinions and satisfaction levels that were expressed, the use and quantification of PTA parameters in the context of feedback made the teams aware of the importance of these aspects in the implementation of a quality policy for practical training. This supports our hypothesis H2.

Secondly, certain very satisfactory results in departments or healthcare units paved the way for best practices, which once identified were promoted and disseminated. By contrast, for unsatisfactory results, it was possible to establish development objectives based on targeted findings. Specifically, the results from the item on energy expenditure during placements proved hard to interpret, which led us to change the wording.

From a medium-term point of view, there are plans to further extend the sharing of mentoring best practices identified in this way. The purpose of this would be to help the departments that need it and promote the most successful mentoring skills. These findings reinforce our hypothesis H3. By way of example, the item on tripartite agreements led to reflection on the teaching practices associated with placements.

In connection with the above and hypothesis H4, a working group was set up as a result of the findings about tripartite agreements, in order to produce recommendations of use to practitioners.

Finally, disseminating and using the results within the departments of the institution allowed staff to ensure that the desired institutional standard of 80% satisfaction was achieved. Results below this level were analysed and gave rise to corrective action. Results within the target served to positively highlight the work of the teams. On the whole, this data provided a kind of “energy” or motivation on the part of those responsible for training (trainers, teams and managers), encouraging them to analyse their student mentoring practices and make changes where appropriate. This third finding also reinforces hypotheses H2, H3 and H4.

In the months that followed the implementation of the CCQCSE model, Swiss Universities of Applied Sciences (UAS) of various sectors showed considerable interest in the results of the survey. Several presentations were therefore given at their request, leading to a great deal of reflection. This reflection revealed a shared dissatisfaction regarding the performance of tripartite agreements. The data produced by the CCQCSE model would thus appear to nurture relations with the institution’s external partners in a very positive way.

While the outcome of implementing the CCQCSE model is very positive, many challenges remain to be addressed following the first deployment of surveys. Firstly, the approach will need to be extended to all students on placements at CHUV and the hospital’s human resources department is currently looking into this.

Useful and informative as it may be, the approach nevertheless came up against a number of limitations. The first, and most significant in our opinion, was the time it took to implement. Specifically, it took more than thirty meetings over the course of the preparation year to design the CCQCSE and implement its processes. The number of people involved in creating the method was also very high. Besides the working group consisting of five people, around 40 participants took part through the control groups. In this regard, the support of the Healthcare Board was key in bringing cycle design efforts to a successful conclusion. All the energy devoted to designing the cycle meant that solid foundations were laid, which now result in timesaving when the cycle is deployed.

## Conclusion and Prospects

Based on the results produced by this project and the first deployment of CCQCSE, it was possible to formalise and quantify the various aspects of the support received by students on placements at CHUV. Increasing the visibility of mentoring actions has thus reinforced the design of practical training while at the same time helping to improve its quality.

Transforming the CCQCST into CCQCSE gave rise to a robust method in line with the quality measures already in place at CHUV. In particular, it encouraged those responsible for the practical training to reflect on their mentoring practices.

Finally, analysing the results allows strengths and weaknesses in the mentoring of placement students to be more accurately diagnosed, if not anticipated. Specifically, over the longer term, by analysing the results it would be possible to highlight aspects of the practical training scheme (PTS) that required work. Our aim over the coming years is to establish this kind of forward-looking perspective within CHUV, with a view to continuously improving the quality of the PTS based on the systematic use of results produced.

## Electronic Supplementary Materials

ESM 1 (DOCX 23 kb)

ESM 2 (PDF 57 kb)

## Footnotes

1 https://www.newsweek.com/2019/04/05/10-best-hospitals-world-1368512.html.

## Abbreviations

CCQCSE: Cycle of Construction and Quality Control for Satisfaction Evaluations
CCQCST: Cycle of Construction and Quality Control for Standardised Testing
CHUV: Lausanne University Hospital
HES-SO: University of Applied Sciences and Arts of Western Switzerland
PTA: Parametrisation of Teaching Actions
PTS: Practical Training Scheme
ToS: Table of Specifications
UAS: Universities of Applied Sciences
UTE Vaud: University of Teacher Education of State of Vaud
