# Supplementary material for: Healthcare Students on Placements: a Cyclical Quality Method for Satisfaction Assessments
Source: Med Sci Educ. 2020 Sep 1;30(4):1427–35. doi: 10.1007/s40670-020-01048-2 (PMC8368566; doi:10.1007/s40670-020-01048-2)
Supplement: Supplementary file 1 — (DOCX 23 kb) [file 40670_2020_1048_MOESM1_ESM.docx]

| **Satisfaction questionnaire** |
| --- |


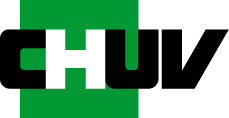


| The information collected will help to improve the supervision of students on internships at the CHUV  Thank you for freely giving your opinion.  For each of the statements, please tick the box corresponding to your appreciation.  Comments can be added at the end of the questionnaire.  Thank you for your collaboration!    **The questionnaire complies with the law on the protection of personal data (LPrD) of 11 September 2007.** |
| --- |

| \| **General information** \| \| --- \| |
| --- | --- |

| **Select the study programme in which you are doing your internship:** |
| --- |
|  |

| **Select your education level:** |
| --- |
| \| 🔾 Health Propaedeutic Year (HPY) \| 🔾 2nd year \| \| --- \| --- \| \| 🔾 1st year \| 🔾 3rd year \| |

| \| **Welcome** \| \| --- \| |
| --- | --- |

| \| **Question 1** \| \| --- \| |
| --- | --- |
| \|  \| Strongly disagree \| Disagree \| Slightly disagree \| Neutral \| Slightly agree \| Agree \| Strongly agree \| \| --- \| --- \| --- \| --- \| --- \| --- \| --- \| --- \| \| I felt welcomed by the staff* \| 🔾 \| 🔾 \| 🔾 \| 🔾 \| 🔾 \| 🔾 \| 🔾 \| |

| \| **Supervision by the staff** \| \| --- \| |
| --- | --- |

| \| **Question 2** \| \| --- \| |
| --- | --- |
| \|  \| Strongly disagree \| Disagree \| Slightly disagree \| Neutral \| Slightly agree \| Agree \| Strongly agree \| \| --- \| --- \| --- \| --- \| --- \| --- \| --- \| --- \| \| I felt helped by the staff*. \| 🔾 \| 🔾 \| 🔾 \| 🔾 \| 🔾 \| 🔾 \| 🔾 \| |
| \| **Question 3** \| \| --- \| |
| \|  \| Strongly disagree \| Disagree \| Slightly disagree \| Neutral \| Slightly agree \| Agree \| Strongly agree \| \| --- \| --- \| --- \| --- \| --- \| --- \| --- \| --- \| \| The atmosphere within the staff* was conducive to my learning. \| 🔾 \| 🔾 \| 🔾 \| 🔾 \| 🔾 \| 🔾 \| 🔾 \| |
| \| **Question 4** \| \| --- \| |
| \|  \| Strongly disagree \| Disagree \| Slightly disagree \| Neutral \| Slightly agree \| Agree \| Strongly agree \| \| --- \| --- \| --- \| --- \| --- \| --- \| --- \| --- \| \| I was able to easily access resources useful to my learning (patient records, protocols, clinical workshops, articles, internet...). \| 🔾 \| 🔾 \| 🔾 \| 🔾 \| 🔾 \| 🔾 \| 🔾 \| |

| **The notion of staff is to be taken in the broadest sense, including all employees (managers, practitioners, trainers, professionals - graduates, support staff, ...).* |
| --- |

| \| **Clinical education by the mentor** \| \| --- \| |
| --- | --- |

| \| **Question 5** \| \| --- \| |
| --- | --- |
| \|  \| Strongly disagree \| Disagree \| Slightly disagree \| Neutral \| Slightly agree \| Agree \| Strongly agree \| \| --- \| --- \| --- \| --- \| --- \| --- \| --- \| --- \| \| The supervision of the mentor facilitated the development of my skills. \| 🔾 \| 🔾 \| 🔾 \| 🔾 \| 🔾 \| 🔾 \| 🔾 \| |

| \| **Question 6** \| \| --- \| |
| --- | --- |
| \|  \| Strongly disagree \| Disagree \| Slightly disagree \| Neutral \| Slightly agree \| Agree \| Strongly agree \| \| --- \| --- \| --- \| --- \| --- \| --- \| --- \| --- \| \| The time provided by the mentor for my supervision was well suited to my needs. \| 🔾 \| 🔾 \| 🔾 \| 🔾 \| 🔾 \| 🔾 \| 🔾 \| |

| \| **Question 7** \| \| --- \| |
| --- | --- |
| \|  \| Strongly disagree \| Disagree \| Slightly disagree \| Neutral \| Slightly agree \| Agree \| Strongly agree \| \| --- \| --- \| --- \| --- \| --- \| --- \| --- \| --- \| \| The theory-practice links highlighted allowed me to develop my skills. \| 🔾 \| 🔾 \| 🔾 \| 🔾 \| 🔾 \| 🔾 \| 🔾 \| |

| \| **Internship environment** \| \| --- \| |
| --- | --- |

| \| **Question 8** \| \| --- \| |
| --- | --- |
| \|  \| Strongly disagree \| Disagree \| Slightly disagree \| Neutral \| Slightly agree \| Agree \| Strongly agree \| \| --- \| --- \| --- \| --- \| --- \| --- \| --- \| --- \| \| Working hours were at the service of my training. \| 🔾 \| 🔾 \| 🔾 \| 🔾 \| 🔾 \| 🔾 \| 🔾 \| |

| \| **Question 9** \| \| --- \| |
| --- | --- |
| \|  \| Strongly disagree \| Disagree \| Slightly disagree \| Neutral \| Slightly agree \| Agree \| Strongly agree \| \| --- \| --- \| --- \| --- \| --- \| --- \| --- \| --- \| \| This internship required a reasonable expenditure of energy. \| 🔾 \| 🔾 \| 🔾 \| 🔾 \| 🔾 \| 🔾 \| 🔾 \| |

| \| **Objectives and Evaluations** \| \| --- \| |
| --- | --- |

| \| **Question 10** \| \| --- \| |
| --- | --- |
| \|  \| Strongly disagree \| Disagree \| Slightly disagree \| Neutral \| Slightly agree \| Agree \| Strongly agree \| \| --- \| --- \| --- \| --- \| --- \| --- \| --- \| --- \| \| The tripartite contract contributed to the development of my professional skills. \| 🔾 \| 🔾 \| 🔾 \| 🔾 \| 🔾 \| 🔾 \| 🔾 \| |

| \| **Question 11** \| \| --- \| |
| --- | --- |
| \|  \| Strongly disagree \| Disagree \| Slightly disagree \| Neutral \| Slightly agree \| Agree \| Strongly agree \| \| --- \| --- \| --- \| --- \| --- \| --- \| --- \| --- \| \| The mid-term and end-of-term evaluations were useful for my progress. \| 🔾 \| 🔾 \| 🔾 \| 🔾 \| 🔾 \| 🔾 \| 🔾 \| |

| \| **Motivation** \| \| --- \| |
| --- | --- |

| \| **Question 12** \| \| --- \| |
| --- | --- |
| \|  \| Strongly disagree \| Disagree \| Slightly disagree \| Neutral \| Slightly agree \| Agree \| Strongly agree \| \| --- \| --- \| --- \| --- \| --- \| --- \| --- \| --- \| \| The internship strengthened my motivation to pursue my professional project. \| 🔾 \| 🔾 \| 🔾 \| 🔾 \| 🔾 \| 🔾 \| 🔾 \| |

| \| **Question 13** \| \| --- \| |
| --- | --- |
| \|  \| Strongly disagree \| Disagree \| Slightly disagree \| Neutral \| Slightly agree \| Agree \| Strongly agree \| \| --- \| --- \| --- \| --- \| --- \| --- \| --- \| --- \| \| I would recommend this internship to other students. \| 🔾 \| 🔾 \| 🔾 \| 🔾 \| 🔾 \| 🔾 \| 🔾 \| |

| \| **Comments** \| \| --- \| |
| --- | --- |

| \| **What are the strong points of this internship ?** \| \| --- \| |
| --- | --- |
|  |

| \| **What are your suggestions to improve this internship ?** \| \| --- \| |
| --- | --- |
|  |
