# Supplementary figures and images for: Healthcare Students on Placements: a Cyclical Quality Method for Satisfaction Assessments
Source: Med Sci Educ. 2020 Sep 1;30(4):1427–35. doi: 10.1007/s40670-020-01048-2 (PMC8368566; doi:10.1007/s40670-020-01048-2)

# Course of the project

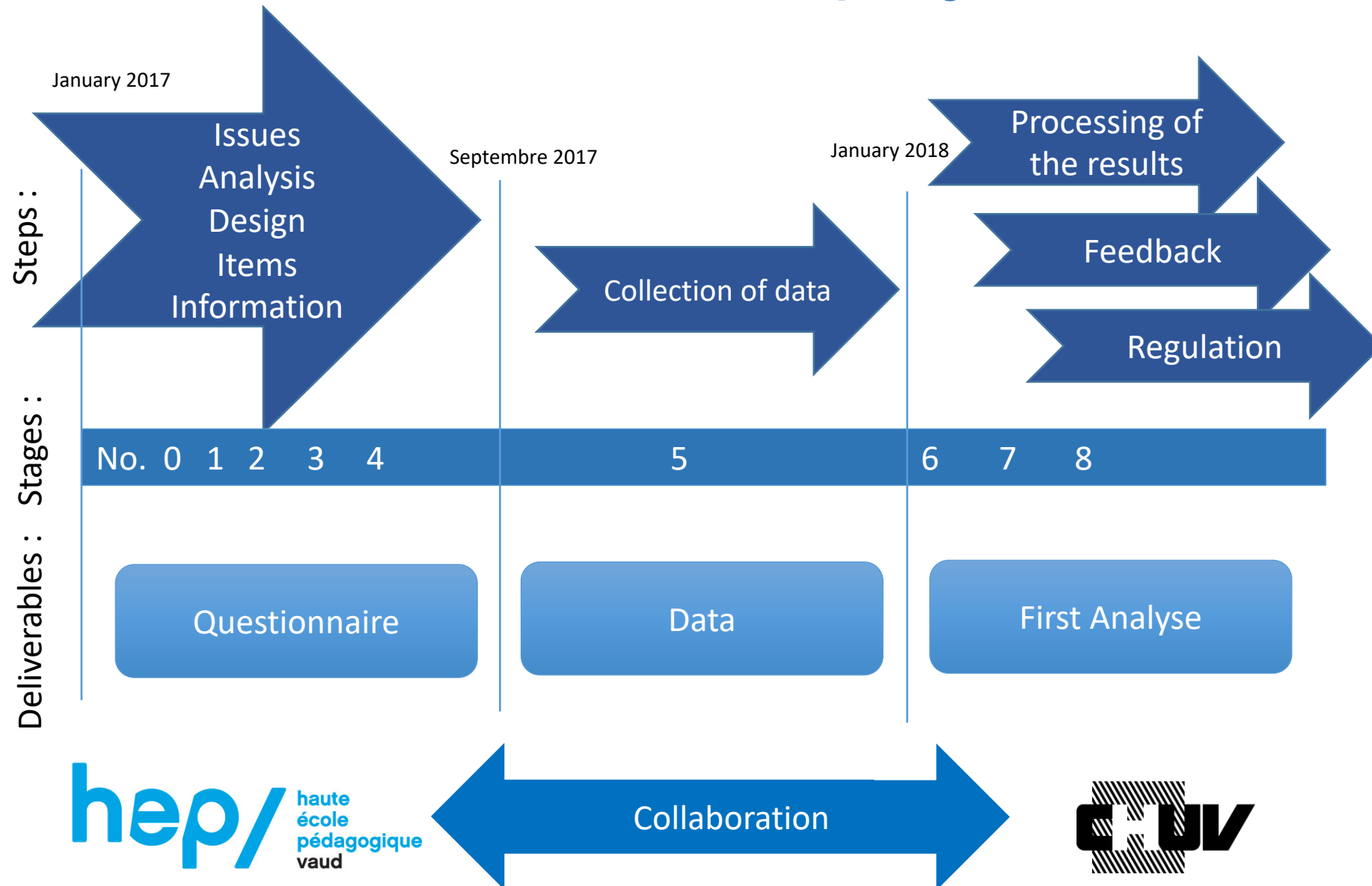

Supplement: Supplementary file 2 — (PDF 57 kb) [file 40670_2020_1048_MOESM2_ESM.pdf]
